# Living With Acne Vulgaris in Young Adults: A Holistic Examination of Its Impact on Quality of Life Using the Dermatology Life Quality Index (DLQI)

**DOI:** 10.7759/cureus.77167

**Published:** 2025-01-08

**Authors:** Venkata Dileep Kumar Veldi, Arun Kumar Metta, Sandhya Metta, Sri Sai Praneeth Angara, Anirudh Srinivas Teja Peela, Sarath Chandra Ponnada

**Affiliations:** 1 Medicine, Gayatri Vidya Parishad Institute of Health Care and Medical Technology, Visakhapatnam, IND; 2 Dermatology, Gayatri Vidya Parishad Institute of Health Care and Medical Technology, Visakhapatnam, IND; 3 Physiology, Gayatri Vidya Parishad Institute of Health Care and Medical Technology, Visakhapatnam, IND; 4 General Medicine, NRI Institute of Medical Sciences, Visakhapatnam, IND; 5 General Medicine, Great Eastern Medical School and Hospital, Srikakulam, IND

**Keywords:** acne vulgaris, dermatology, dlqi, psychosocial impact, quality of life, young adults

## Abstract

Background: Acne vulgaris is an inflammatory skin disease that shows chronic effects in adolescents and young adults. Its impact on quality of life (QoL) often extends beyond the physical symptoms, influencing social interactions and reducing self-confidence. Although these impacts are widely acknowledged, they are seldom given sufficient attention or assessed in depth with standardized measures like the Dermatology Life Quality Index (DLQI). This study aimed to evaluate the extent to which acne interferes with QoL in young adults using DLQI scores and its associations with demographic and clinical factors.

Methods: This hospital-based, cross-sectional study was conducted between October 2023 and December 2023 at Gayatri Vidya Parishad Institute of Health Care and Medical Technology, Visakhapatnam, India. The study population comprised 200 participants aged 16-28 diagnosed with acne. Data collected included demographic factors (age, sex), clinical features (acne grade, duration, site, scars, hyperpigmentation, and skin type), and DLQI scores. Acne severity was classified from grade I to IV, and the relationships between DLQI scores and clinical/demographic variables were analyzed using chi-squared tests, with statistical significance set at p<0.05. Data analysis was performed using IBM SPSS Statistics for Windows, Version 26.0 (Released 2019; IBM Corp., Armonk, New York, United States).

Results: The mean age of participants was 19.6 years, with women comprising 68.5% of the study population. Acne grade II was the most common, and the average DLQI score was 11.14, indicating a noticeable impact on QoL. Nearly half of the participants (48%) reported a "very large" effect on QoL. Highly significant associations were found between duration of acne, post-acne hyperpigmentation, and grade of acne with DLQI scores (all p<0.001), showing that longer duration, hyperpigmentation, and higher grades of acne significantly worsened QoL. Significant associations were also observed for site of acne and acne scars (both p<0.05), with multiple acne sites and severe scarring linked to greater negative impacts on DLQI scores. Grade III-IV acne were especially tied to bigger hits on QoL. Additionally, site of acne and acne scars were highly associated with acne grade (p<0.001). Factors such as having acne for over two years, oily skin, acne across multiple sites (face, chest, and back), and post-acne hyperpigmentation contributed to higher DLQI scores.

Conclusion: Acne vulgaris has a marked impact on QoL in young adults, especially those with severe, persistent, and extensive cases. Focusing on both the physical and mental aspects of acne in treatment could make a difference. Further multi-center studies with larger samples are recommended to generalize these findings and help create targeted interventions.

## Introduction

Acne vulgaris, a prevalent chronic inflammatory disorder of the pilosebaceous unit, typically emerges during adolescence due to *Cutibacterium acnes* and fluctuating levels of dehydroepiandrosterone (DHEA). It manifests as both inflammatory and non-inflammatory lesions, more predominantly on the face, but also affecting the upper body [1]. Although traditionally considered a cosmetic issue, the significant negative psychosocial effects of acne, like reduced self-esteem and heightened social anxiety, are now well-documented, with studies showing improvements in these areas following effective treatment [2]. Despite this, there remains a critical need for comprehensive assessments of acne's impact on overall well-being, particularly through quality of life (QoL) measures like the Dermatology Life Quality Index (DLQI). Dermatological conditions, including acne, can profoundly affect mental health, self-confidence, and social interactions, making the assessment of QoL an essential component of patient care [3,4]. QoL, as defined by the World Health Organization (WHO), refers to individuals' perceptions of their position in life within the context of the culture, value systems, goals, expectations, standards, and concerns they encounter [5]. The DLQI, a validated tool for measuring QoL in dermatology patients, offers valuable insights into how acne impacts daily living, yet its use in evaluating the holistic burden of acne vulgaris is relatively underexplored. The global prevalence of acne among adolescents underscores its significance as a public health concern, with rates ranging from 28.9% to 91.3% [6]. In Asian populations, acne prevalence has been reported at 33% in China, 34% in Malaysia, and 56% in Saudi Arabia [7-9]. Similarly, in African countries such as Nigeria and Egypt, the prevalence exceeds 60% among female adolescents [10,11]. These statistics highlight the need for further research into the psychosocial impact of acne, particularly during the formative years of adolescence. Accurate grading of acne is crucial for assessing its severity and guiding treatment, as emphasized by Adityan et al., who analyzed standardized scoring systems for acne vulgaris [12]. This study aimed to fill the research gap by using the DLQI to assess how acne vulgaris affects the QoL in young adults. By shedding light on the daily challenges experienced by those with acne, this research hopes to guide the development of more personalized and effective dermatological treatments.

Objectives

This study aimed to estimate the levels of QoL among the patients affected with acne and to determine the association between demographic and clinical characteristics of acne and the levels of QoL.

## Materials and methods

Study type, setting, and duration

This observational, cross-sectional, hospital-based study was conducted at Gayatri Vidya Parishad Institute of Health Care and Medical Technology, Visakhapatnam, India, between October 2023 and December 2023.

Sample size

**Figure 1:**
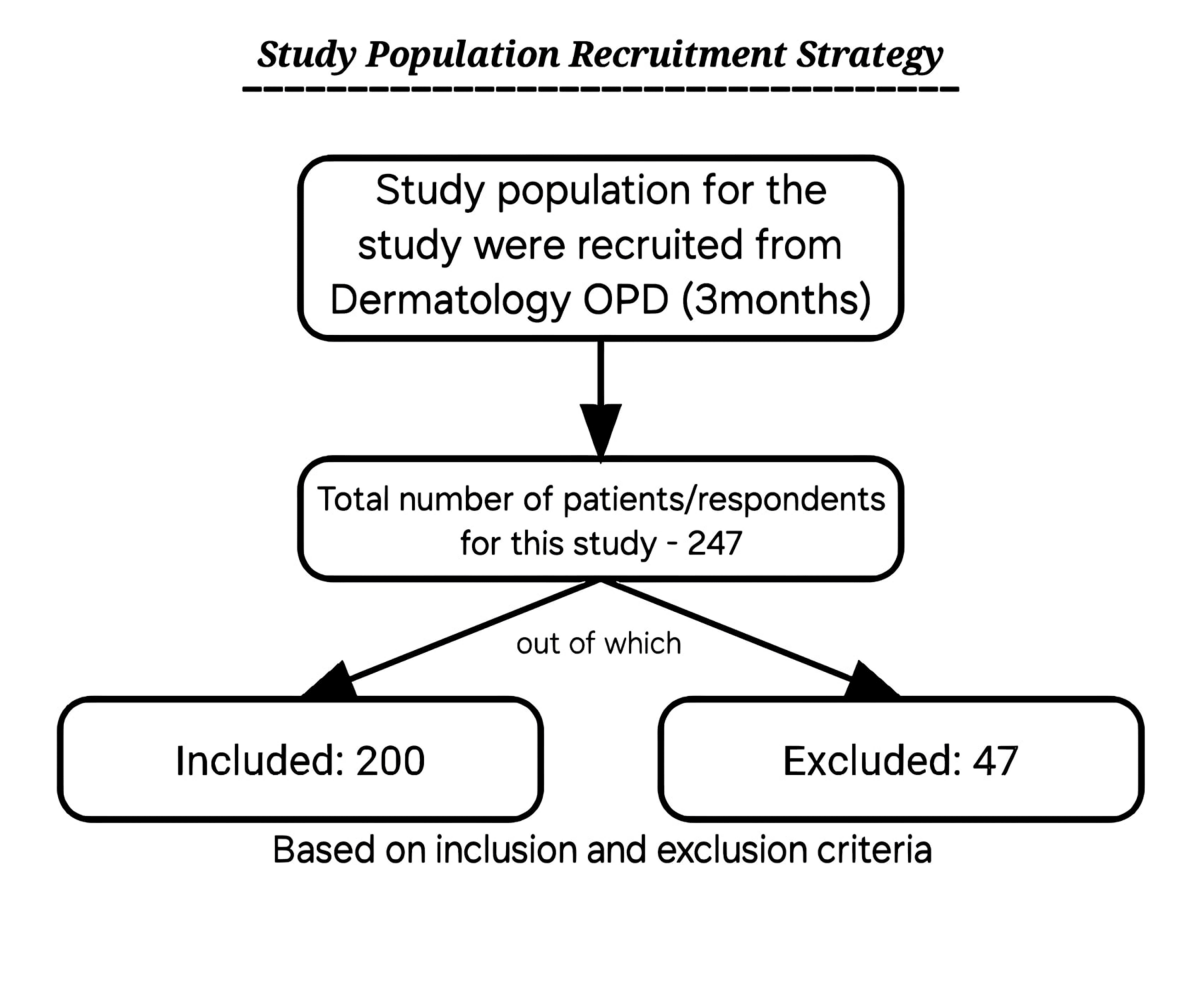
Flowchart for sample recruitment

As this study was done as an Undergraduate Student Research Scholarship (UGSRS) project, the study duration was three months. We recruited the study population based on the purposive sampling technique. From the dermatology outpatient department, a total of 247 respondents were recruited, of which 200 patients were included and 47 were excluded based on the inclusion and exclusion criteria (Figure 1).

Study tool

A standard DLQI questionnaire was used to assess the QoL of patients.

Inclusion criteria

Included were patients diagnosed with acne aged between 16 and 28 years and patients who agreed to take written voluntary informed consent after the questionnaire was explained to them.

Exclusion criteria

Excluded were patients with acne who have a documented history of disabilities that can affect their mental state and use of topical and systemic drugs that aggravate acne and steroid-induced acne.

Methods

Clinicodemographic data, including age, sex, type of skin, duration, site, grade of acne, post-acne hyperpigmentation, and acne scars, was collected in a well-designed proforma (Appendix 1). The grading of acne was taken from standardized scoring systems [12]. Moreover, acne severity was classified as follows: grade I: comedones and occasional papules; grade II: papules, comedones, and few pustules; grade III: predominant pustules, nodules, and abscesses; and grade IV: mainly cysts, abscesses, and widespread scarring. The relationship between acne vulgaris and its sequelae was analyzed using the DLQI questionnaire [13], which includes 10 domains assessing various aspects of daily life impacted by acne and its score grading. The DLQI questionnaire was provided in Appendix 2.

Ethical consideration

Approval from the Institutional Ethics Committee of Gayatri Vidya Parishad Institute of Health Care and Medical Technology was secured before the start of the study, with the approval number GVPIHCMT/IEC/20231031/03. The DLQI questionnaire was administered by trained medical students during routine outpatient visits, supervised by a dermatologist. Participants received a detailed explanation for clarity, and those needing extra guidance were helped step by step. The straightforward language of the questionnaire minimized language barrier issues.

Statistics and data analysis

The data collected was uploaded into Microsoft Excel (Microsoft Corporation, Redmond, Washington, United States) for initial compilation and cleaning. Statistical analysis was carried out using IBM SPSS Statistics for Windows, Version 26.0 (Released 2019; IBM Corp., Armonk, New York, United States). To outline the demographic and clinical features of the study population, descriptive statistics were used.

The DLQI scores were associated with demographic and clinical variables such as age, sexuality, duration of acne, site of acne, grade of acne, presence of post-acne hyperpigmentation, and acne scars. Also, grades of acne were associated with demographic and clinical variables.

A chi-squared test was conducted to examine the relationship between DLQI scores and various demographic and clinical variables, as well as the relationship between the grades of acne and these same variables. A p-value of <0.05 was considered statistically significant, while a p-value of <0.001 was considered statistically highly significant.

## Results

The study included 200 out of 247 patients who came to the outpatient department of dermatology with symptoms of acne, of which 47 patients were excluded for not meeting the inclusion criteria over a period of three months, with a mean age of 19.62 years. The study population was predominantly women, with 137 (68.5%) women (mean age: 19.58) and 63 (31.5%) men (mean age: 19.69). Most participants (153 out of 200) were in the 16-20-year age range, and the majority were unmarried (184 out of 200). Grade II acne was the most common severity observed (Table 1).

**Table 1:** DLQI interpretation DLQI: Dermatology Life Quality Index

DLQI interpretation	No of patients (%)
No effect	0
Mild effect	14 (7)
Moderate effect	81 (40.5)
Very large effect	96 (48)
Extremely large effect	9 (4.5)
Total	200

**Figure 2:**
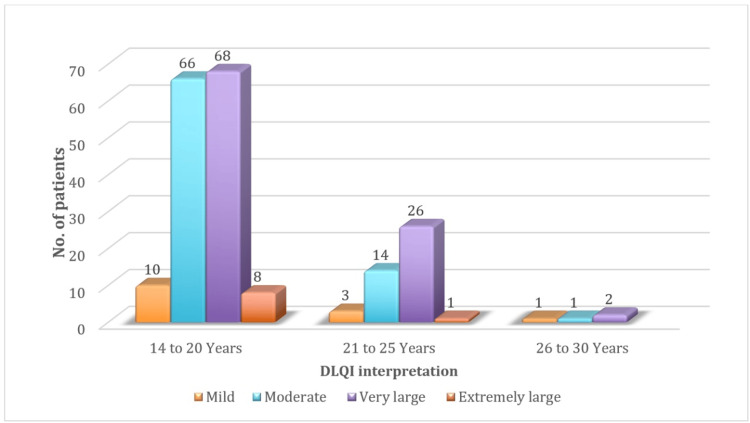
Age distribution with corresponding DLQI categories, highlighting the impact of dermatological conditions on quality of life across age groups DLQI: Dermatology Life Quality Index

**Figure 3:**
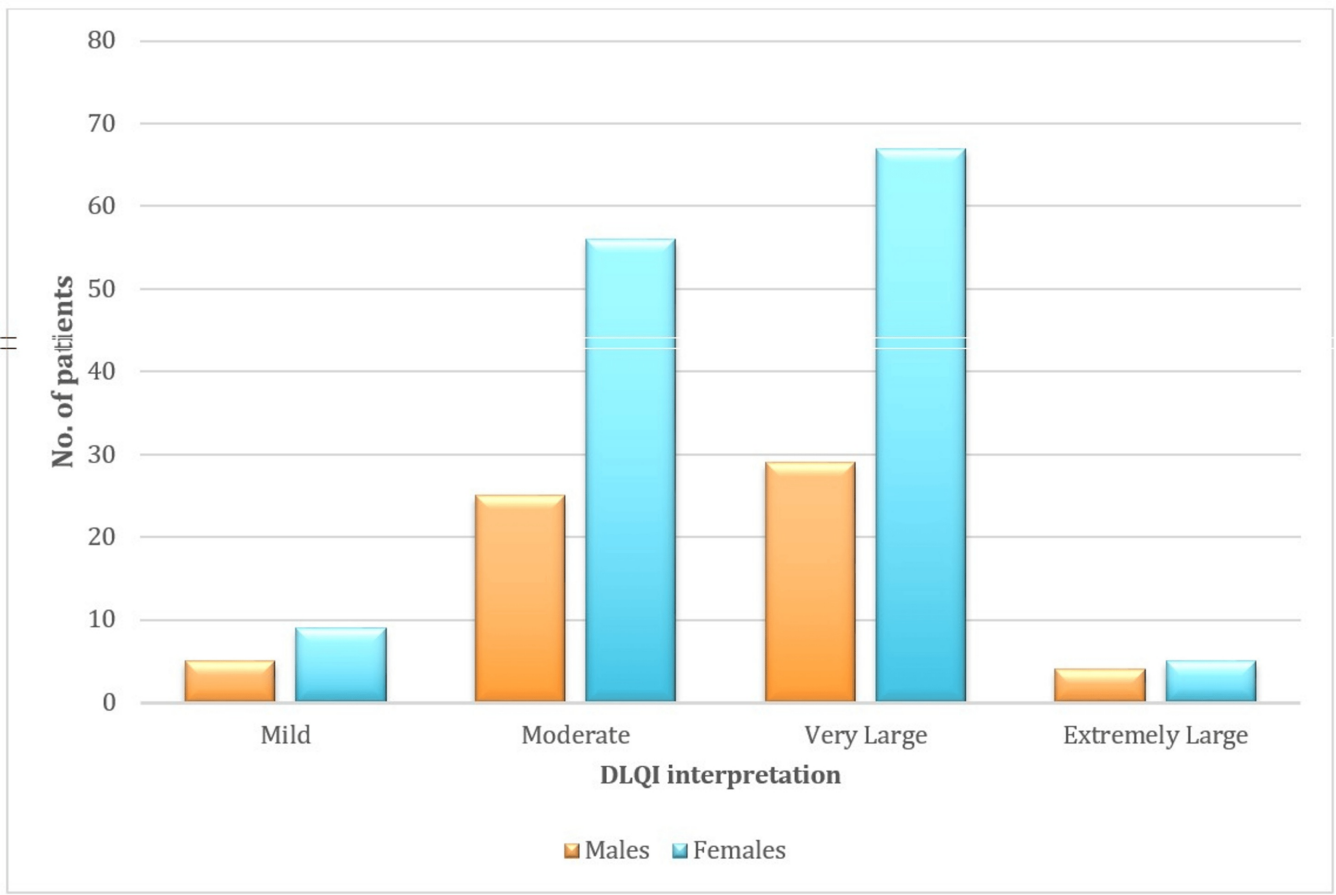
Sex distribution and DLQI categories, illustrating the impact of dermatological conditions on quality of life by gender DLQI: Dermatology Life Quality Index

The mean DLQI score among the participants was 11.14. The distribution of DLQI scores showed a significant impact on the participants' QoL, with scores ranging from mild to extremely large effects on their lives. Most of the participants 96 (48%) reported a "very large" impact on their QoL due to acne (Figure 2 and Figure 3).

**Figure 4:**
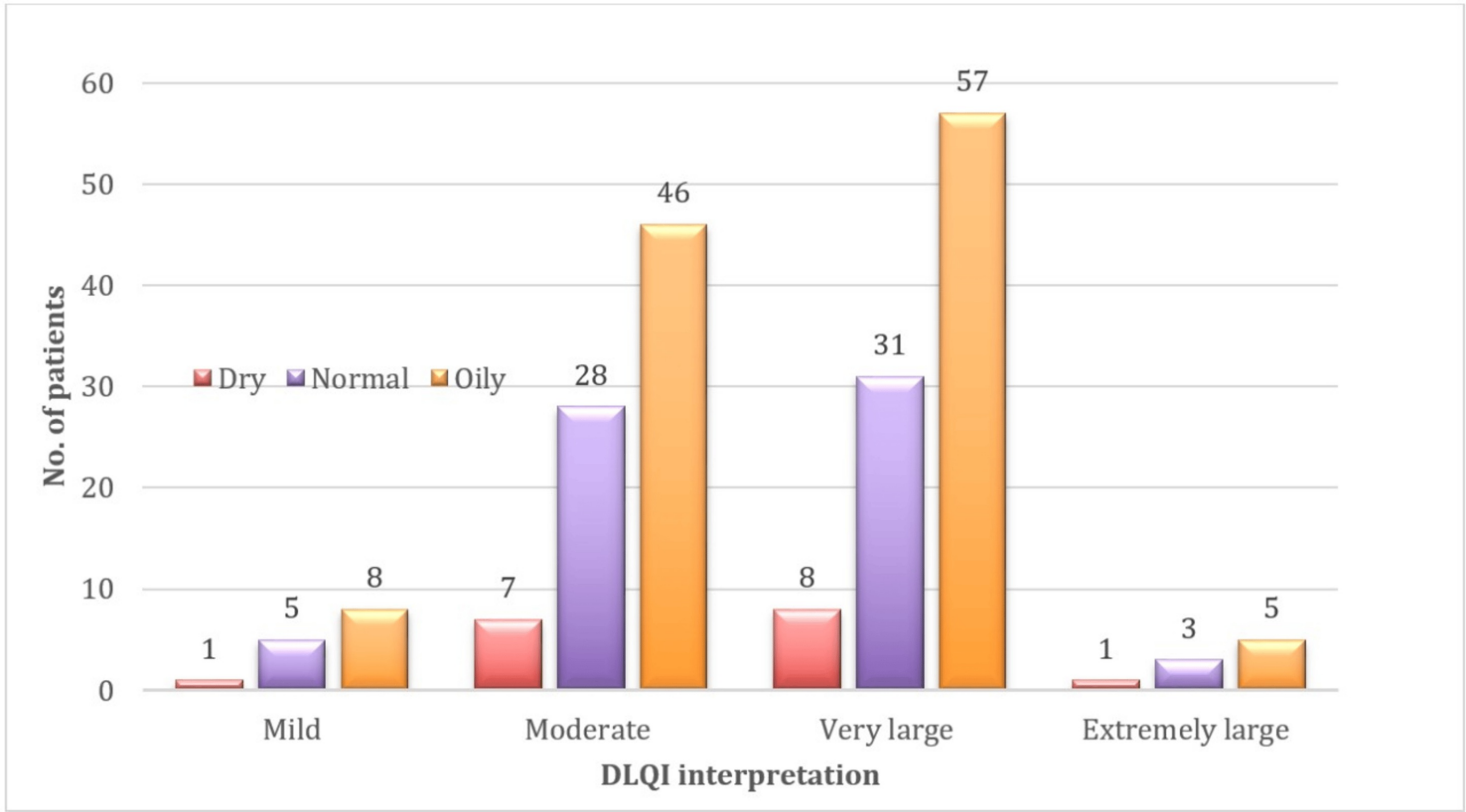
Skin type and DLQI categories, highlighting the impact of dermatological conditions on quality of life DLQI: Dermatology Life Quality Index

Among the 200 participants, 116 (58%) had oily skin, 67 (33.5%) had normal skin, and 17 (8.5%) had dry skin. A "very large" impact on QoL was most common in those with oily skin (57, 28.5%), followed by normal skin (31, 15.5%) and dry skin (8, 4%). Overall, oily skin was linked to a greater negative impact on QoL. Participants with oily skin have higher grades of acne (Figure 4). 

The duration of acne was highly significantly associated with DLQI scores (p<0.001). Participants with a longer duration of acne (12-36 months) tended to report higher DLQI scores, indicating a greater impact on their QoL.

The site of acne also had a statistically significant association with DLQI scores (p<0.05). Participants with acne on the face, chest, and back reported higher DLQI scores compared to those with acne, solely on the face. The site of acne had a highly significant association with the grade of acne (p<0.001). Acne predominantly located on the face was mostly associated with grade II acne. Participants with acne on their face and the back or face, chest, and back had a higher proportion of grade III and IV acne.

The duration of acne had a statistically highly significant association with the grade of acne (p<0.001). The majority of participants with acne lasting 0-6 months had grade II acne. Those with a duration of 6-12 months also mostly had grade II acne but with a higher proportion of grade III and IV cases (Table 2 and Table 3). 

**Table 2:** DLQI and its association with clinical and demographic variables Pearson's chi-squared test; p<0.001: highly significant; p<0.05: significant DLQI: Dermatology Life Quality Index

Category and variables	DLQI interpretation				Chi-squared value	P-value
	Mild	Moderate	Very large	Extremely large		
Duration of acne						
0-6 months	10	47	29	2	29.61	<0.001
6-12 months	2	27	35	2		
12-36 months	2	7	32	5		
Site of acne						
Face	11	65	47	3	25.01	<0.05
Face and back	1	6	17	2		
Face and chest	1	5	22	2		
Face, chest, and back	1	5	10	2		
Acne scars						
Absent	6	25	23	1	22.91	<0.05
Mild	4	30	16	3		
Moderate	2	17	25	1		
Severe	2	9	32	4		
Post-acne hyperpigmentation						
Absent	11	55	40	2	19.24	<0.001
Present	3	26	56	7		

**Table 3:** Grade of acne and its association with clinical and demographic variables Pearson's chi-squared test; p<0.001: highly significant; p<0.05: significant DLQI: Dermatology Life Quality Index

Category and variables	Grade of acne				Chi-squared value	P-value
	I	II	III	IV		
Age range (in years)						
14-20	21	84	38	9	3.86	0.694
21-25	6	21	12	5		
26-30	1	3	0	0		
Sex						
Female	18	73	37	9	1.08	0.780
Male	10	35	13	5		
Duration of acne						
0-6 months	25	52	10	1	68.97	<0.001
6-12 months	1	45	16	4		
12-36 months	2	11	24	9		
Type of skin						
Dry	4	6	5	2	6.03	0.419
Normal	9	37	14	7		
Oily	15	65	31	5		
Site of acne						
Face	21	80	21	4	41.55	<0.001
Face and back	2	11	11	2		
Face and chest	2	12	14	2		
Face, chest, and back	3	5	4	6		
Acne scars						
Absent	17	31	6	1	36.79	<0.001
Mild	8	38	19	4		
Moderate	2	28	12	3		
Severe	1	11	13	6		
Post-acne hyperpigmentation						
Absent	27	67	14	1	49.14	<0.001
Present	1	41	36	13		

**Figure 5:**
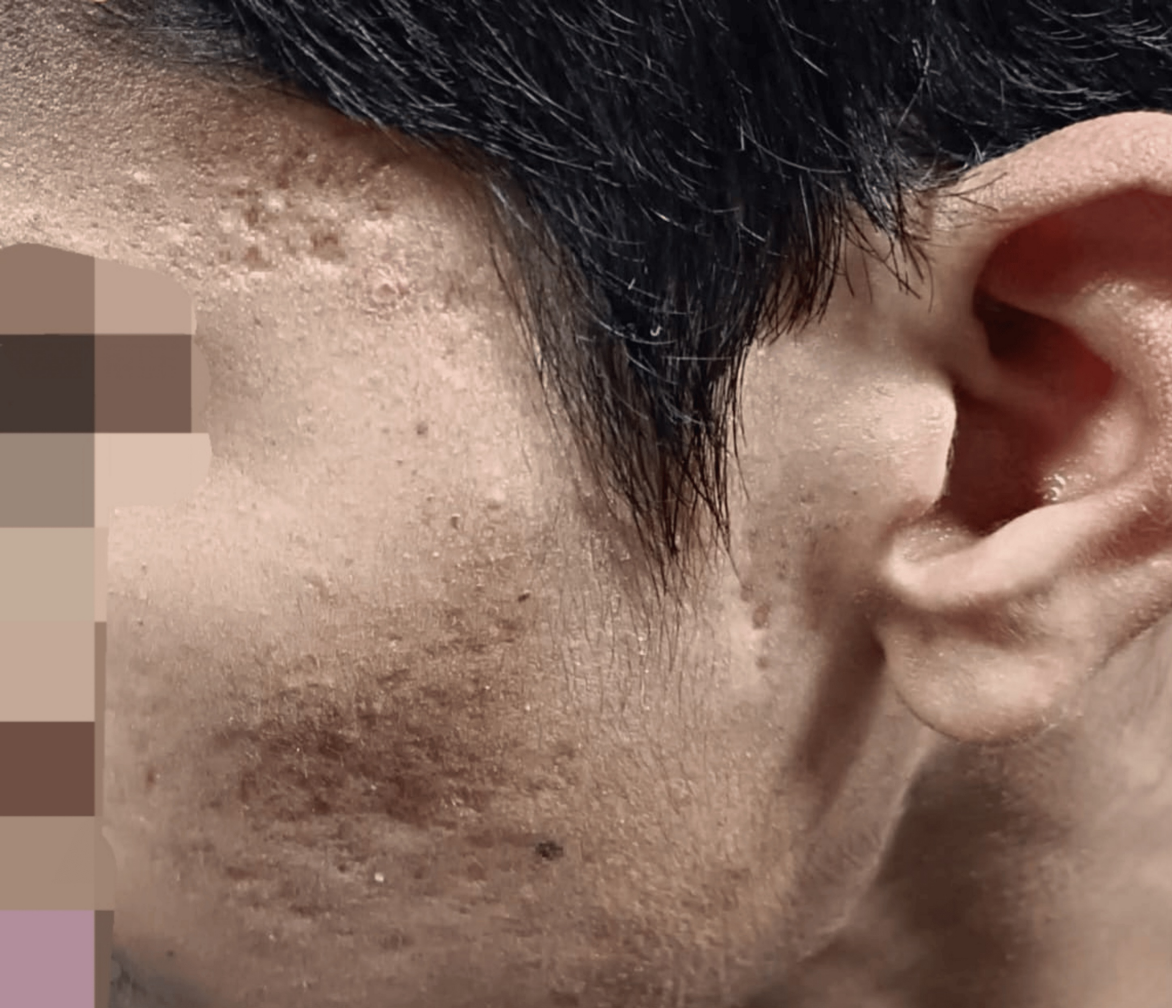
Acne scars Images of patients included in the study, with informed consent for publication

The presence of acne scars and DLQI scores were statistically more significant (p<0.05). Participants with severe acne scars reported higher DLQI scores. The presence of acne scars showed a highly significant association with the grade of acne (p<0.001). Participants with mild scars were mostly in grade II, while those with moderate and severe scars were more evenly distributed across grades II, III, and IV (Figure 5).

**Figure 6:**
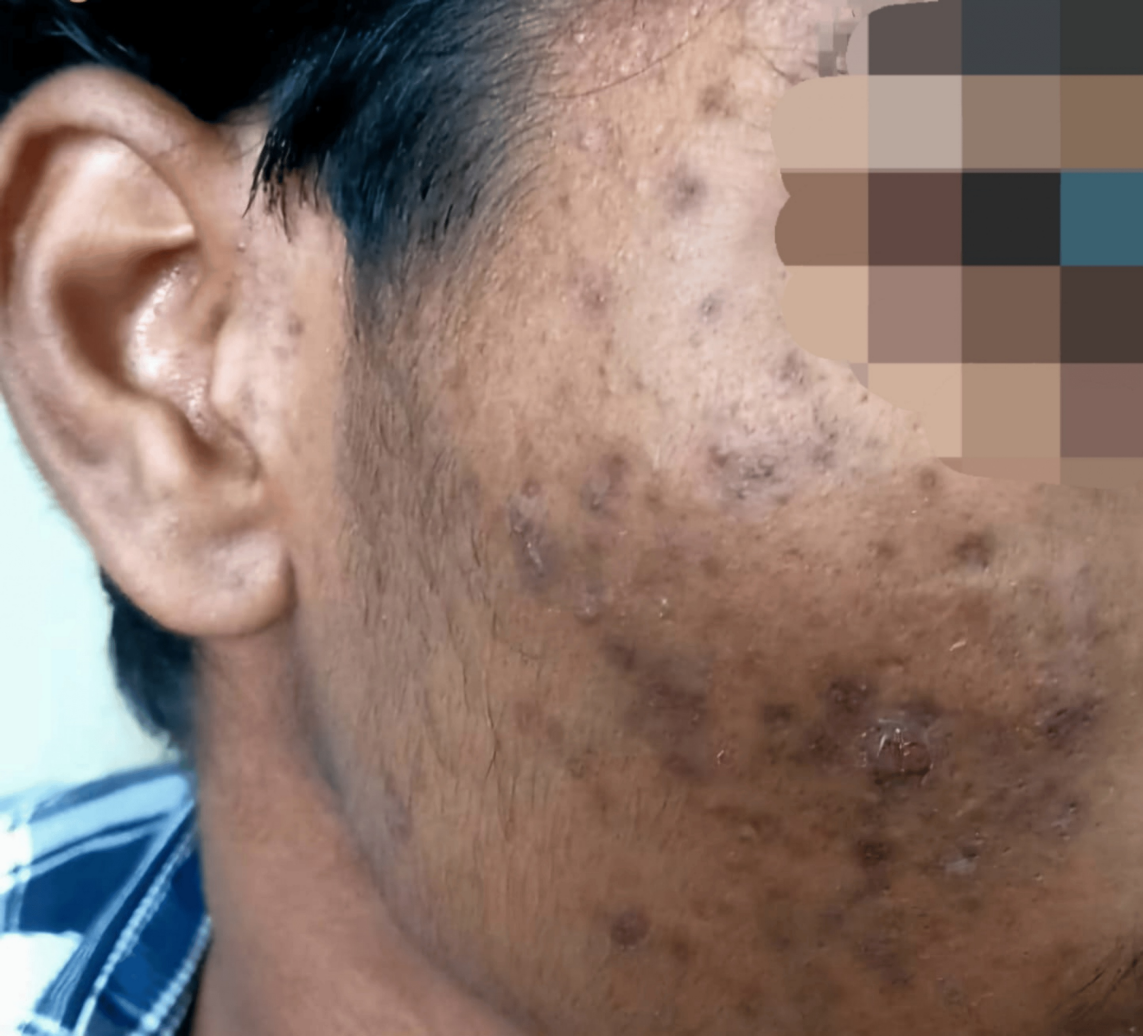
Post-acne hyperpigmentation Images of patients included in the study, with informed consent for publication

The presence of post-acne hyperpigmentation showed a highly significant association with DLQI scores (p<0.001). Participants with post-acne hyperpigmentation reported higher DLQI scores compared to those without. The presence of post-acne hyperpigmentation showed a highly significant association with the grade of acne (p<0.001). Participants without post-acne hyperpigmentation were predominantly in grade II, while those with hyperpigmentation were distributed across grades II, III, and IV (Figure 6).

The DLQI vs. grade table demonstrates the association between DLQI scores and the clinical severity of acne. The data indicated a statistically more significant association (p<0.001) between the grade of acne and DLQI scores. Participants with higher grades of acne reported higher DLQI scores, reflecting a higher impact on their QoL (Table 4).

**Table 4:** DLQI scores and grade of acne Pearson chi-squared test; p<0.001: highly significant; p<0.05: significant DLQI: Dermatology Life Quality Index

Grade of acne	DLQI interpretation						
	Mild	Moderate	Very large	Extremely large	Grand total	Chi-squared value	P-value
I	7	15	5	1	28	80.53	<0.001
II	4	60	42	2	108		
III	2	3	43	2	50		
IV	1	3	6	4	14		
Grand total	14	81	96	9	200		

**Figure 7:**
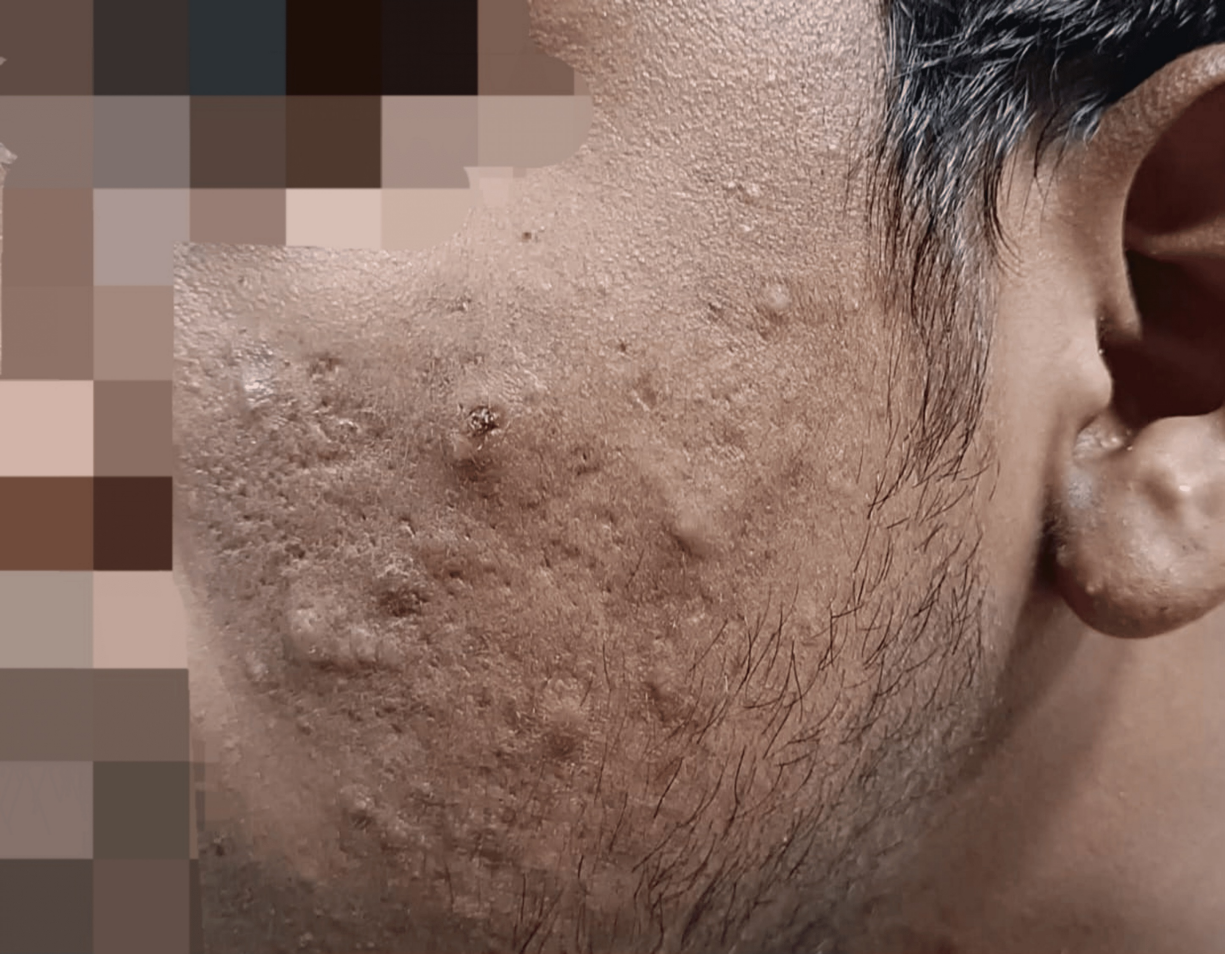
Grade IV acne Images of patients included in the study, with informed consent for publication

Mild DLQI interpretation was predominantly observed in participants with grade I acne, while moderate DLQI scores were most common among those with grade II acne. Participants with grade III acne frequently disclosed a very much impact on the QoL, as indicated by their DLQI scores. The most severe impact, reflected by extremely large DLQI scores, was more commonly associated with participants suffering from grade IV acne (Figure 7).

**Figure 8:**
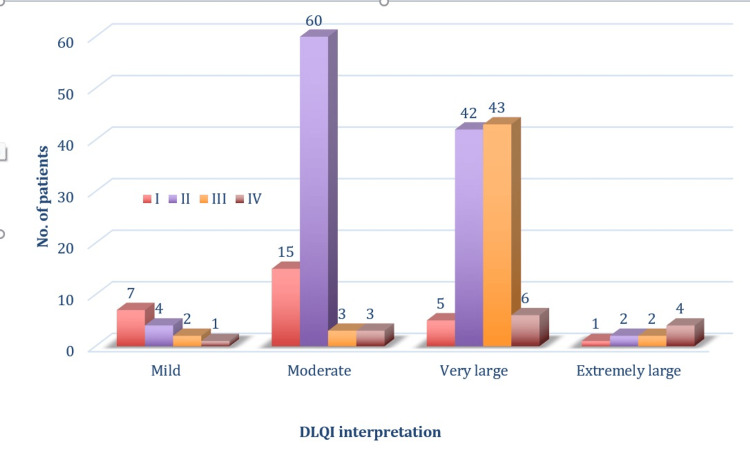
DLQI categories across acne grades, reflecting the impact of acne severity on quality of life DLQI: Dermatology Life Quality Index

In conclusion, the study found that the severity of acne, as measured by the grade of acne, significantly impacts the QoL of young adults, as indicated by the DLQI scores (Figure 8).

## Discussion

The study included a total of 200 participants over a period of three months. In comparison, the hospital-based research by Hazarika and Rajaprabha [14] with self-reported cases of acne vulgaris included 114 participants over six months, and the study by Durai and Nair [15] involved 140 participants over five months.

Our study found a mean DLQI score of 11.14, indicating a significant impact on QoL. This finding is consistent with Hazarika and Rajaprabha [14] and Sivaramakrishnan and Thomas [16], who reported mean DLQI scores of 10.73 and 11.30, respectively. These studies suggest that acne has a substantial impact on patients' lives across different populations.

The mean age of participants in our study was 19.62 years. Younger participants, particularly those aged 16-20 years, reported higher DLQI scores, aligning with findings from Sivaramakrishnan and Thomas [16], who noted that younger patients, particularly those aged 15-20 years (53%), are more negatively impacted by acne. This is likely due to the social pressures and psychological stress associated with adolescence. However, Durai and Nair [15] reported a mean age of 21.46±2.81 years with no significant age-related differences, suggesting that the impact of acne on QoL may be less influenced by age in their study population.

Our study found no significant association between age and acne severity (p=0.694), consistent with findings from Hazarika and Rajaprabha [14] and Sivaramakrishnan and Thomas [16], who reported no significant effect of age on acne severity. However, Chowdary et al. [17] observed a slight trend toward more severe acne in older adolescents, possibly due to population or geographic differences.

Women constituted 68.5% of our participants and had higher mean DLQI scores (11.56) compared to men, who made up 31.5% of the participants with a mean score of 10.52. This gender disparity aligns with findings from Chowdary et al. [17], who reported mean DLQI scores of 8.02 for women and 7.82 for men, likely due to societal pressures and beauty standards. However, Kundale et al. [18] found no statistically significant association between gender and DLQI scores (p=0.752), possibly due to differences in cultural factors or sample size.

Our study also found no significant association between sex and acne severity (p=0.780), with both men and women predominantly having grade II acne. This aligns with findings from Durai and Nair [15] and Hazarika and Rajaprabha [14], who similarly found no association between gender and acne severity. However, Hazarika and Rajaprabha [14] noted that women may experience greater psychological impact despite similar clinical severity.

The duration of acne showed a strong association with higher DLQI scores (p<0.001), highlighting a greater impact on QoL with prolonged acne. This finding is consistent with Hazarika and Rajaprabha [14], who also reported a significant relationship between acne duration and DLQI scores (p<0.05). Similarly, acne duration was significantly associated with severity (p<0.001), a trend supported by Hazarika and Rajaprabha [14], Sivaramakrishnan and Thomas [16], and Chowdary et al. [17]. These results emphasize the importance of early intervention to prevent worsening of the condition.

Participants with oily skin reported a greater QoL impact, with higher proportions of "very large" and "extremely large" DLQI scores. Although no overall association was found between skin type and DLQI scores, this finding aligns with Hazarika and Rajaprabha [14], who reported a significant association (p<0.001) between oily skin and higher DLQI scores. Additionally, oily skin was associated with more severe acne, consistent with Durai and Nair [15] and Hazarika and Rajaprabha [14], who linked oily skin to increased sebum production and acne severity.

The site of acne lesions was significantly associated with DLQI scores (p<0.05). Participants with acne on the face, chest, and back reported higher scores compared to those with facial acne alone. This contrasts with findings from Hazarika and Rajaprabha [14] and Durai and Nair [15], who found no association between the site of acne and QoL. These differences may be due to variations in study populations or regional factors.

A strong statistical relationship was found between acne scars and DLQI scores (p=0.05), with severe scarring associated with higher DLQI scores. This aligns with findings from Hazarika and Rajaprabha [14], where a significant association was reported between scarring and QoL (p<0.05). Furthermore, acne severity was highly associated with scarring (p<0.001), consistent with Durai and Nair [15] and Sivaramakrishnan and Thomas [16], who emphasized the need for early treatment to minimize scarring.

Post-acne hyperpigmentation was significantly related to DLQI scores (p<0.001), reflecting its negative impact on self-esteem. This finding is comparable with Hazarika and Rajaprabha [14], who also reported a significant association (p<0.001) between hyperpigmentation and DLQI scores. Additionally, hyperpigmentation was strongly associated with acne severity (p<0.001), consistent with studies by Durai and Nair [15], Hazarika and Rajaprabha [14], and Chowdary et al. [17].

Grade II acne was the most common severity in our study. We found a significant association between acne severity and DLQI scores (p<0.001), with higher severity grades associated with a greater QoL impact. This finding aligns with studies by Sivaramakrishnan and Thomas [16], Hazarika and Rajaprabha [14], Durai and Nair [15], Kundale et al. [18], and Chowdary et al. [17].

Our study adds to the existing evidence highlighting the complex and multifaceted impact of acne on QoL including the psychological impact, mental health support, strong association with higher DLQI scores, and clinical similarities across groups. By aligning our results with previous research, we emphasize the importance of early, personalized, and holistic approaches to acne management. These insights can help clinicians enhance patient care and improve health outcomes for those living with acne.

Limitations

While this study offers valuable insights into the psychosocial burden of acne, it is important to acknowledge certain limitations. Since this is a cross-sectional study, we were unable to draw firm conclusions about cause-and-effect relationships between clinical factors and QoL. The relatively small sample size and the short duration of the study (three months) may have limited the scope of our findings. Although our focus on a single center allowed for consistency, it may also reduce how broadly the results can be applied to other populations. We recognize that important factors such as diet, stress, hormonal influences, and previous treatment history were not assessed, which could have further enriched the analysis. Additionally, as with many studies relying on self-reported data, there is the possibility of recall bias, and the participant pool may reflect certain selection biases. Despite these limitations, we believe this study provides meaningful contributions to understanding how acne affects young adults, and we hope it can serve as a foundation for future research.

## Conclusions

Acne vulgaris is the most common skin problem among adolescents and adults. Acne is not just a dermatological problem or a cosmetic problem but also impacts the psychological health of many affected persons. It is of different grades, occurring in different sites of the body, affecting different skin types, and lasting for different durations, and acne vulgaris affects the QoL of the individual. A longer acne duration is associated with higher DLQI scores and more severe cases. Oily skin showed a greater negative impact, and the acne site significantly influenced DLQI scores, particularly for facial acne. The study showed that there exists a significant association between acne scars and post-acne hyperpigmentation with DLQI. Higher DLQI scores were associated with higher-graded acne scars and greater grades of acne. Most patients scoring mild scars were in grade II, whereas moderate and severe scars range from grades II to IV. Post-acne hyperpigmentation also showed a strong connection with DLQI scores, as people with hyperpigmentation reported higher impacts on QoL. The results showed that the more severe grades of acne lead to increased DLQI scores, which demonstrated an important effect on young adults' lives.

## Figures and Tables

**Figure 9:**
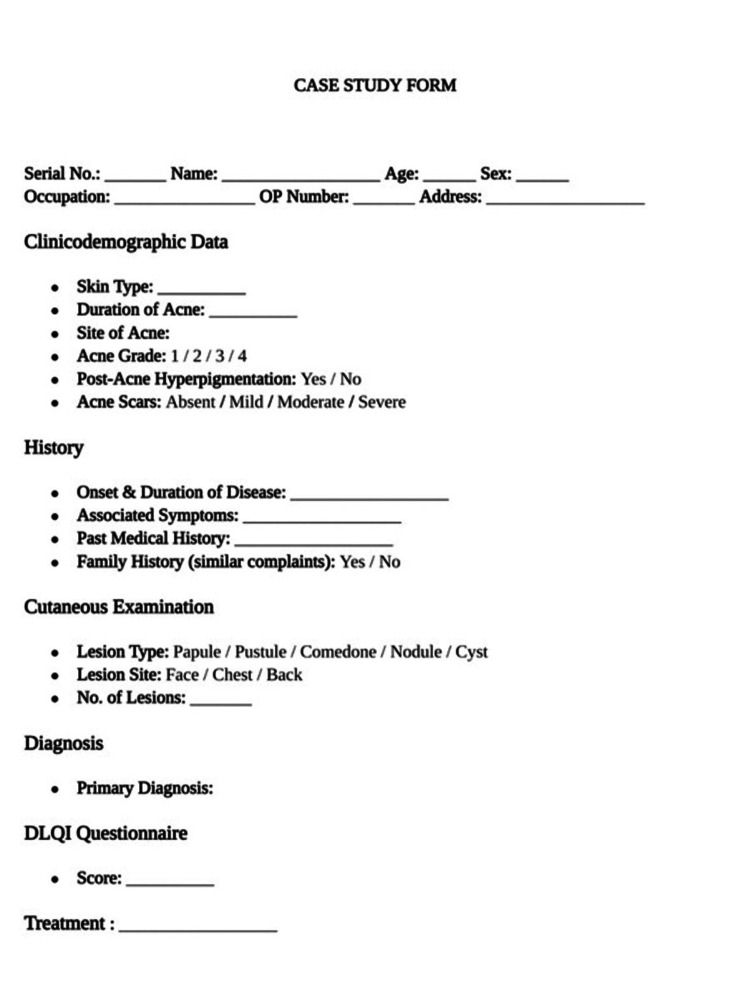
Case study form

**Figure 10:**
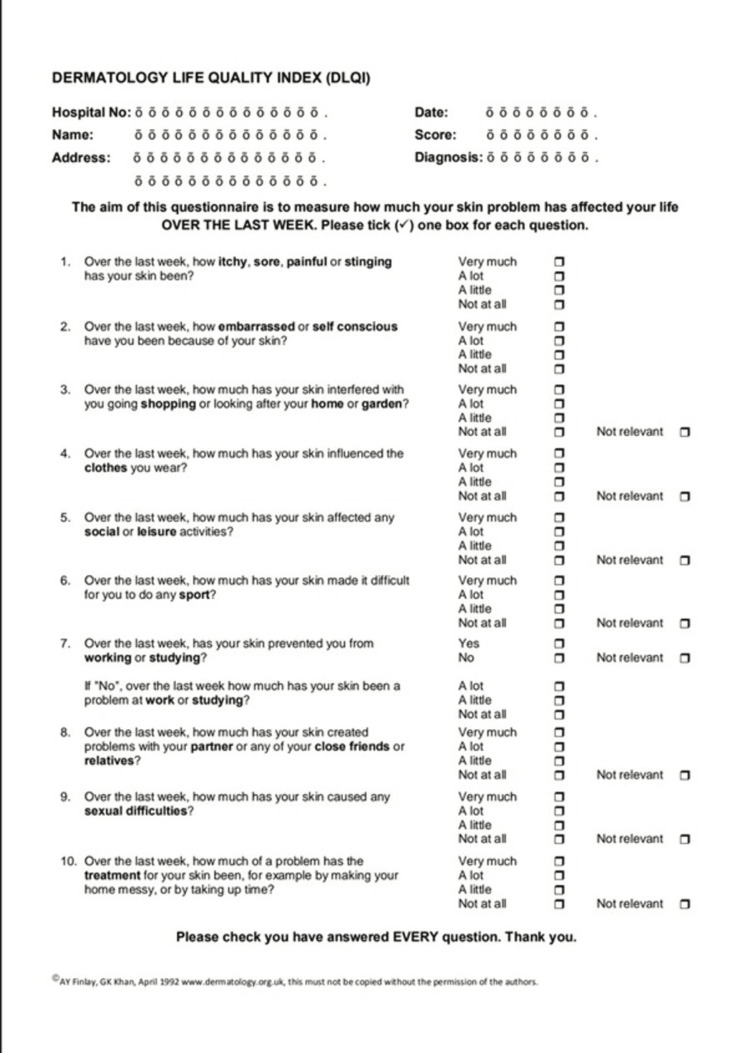
DLQI questionnaire Image Source: Finlay and Khan, 1994 [13]. We have obtained permission from the original publisher "Oxford University Press and Copyright Clearance Center" DLQI: Dermatology Life Quality Index

**Figure 11:**
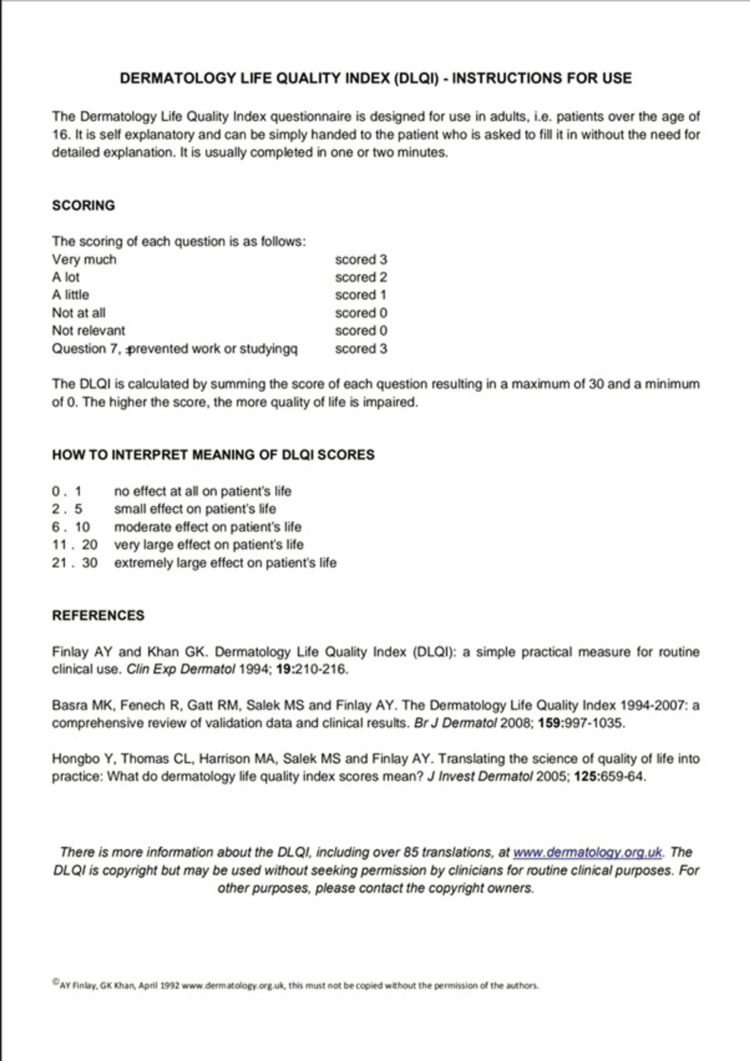
DLQI questionnaire (Continued) Image Source: Finlay and Khan, 1994 [13]. We have obtained permission from the original publisher "Oxford University Press and Copyright Clearance Center" DLQI: Dermatology Life Quality Index

## Appendices

Appendix 1

Appendix 2
